# 

**Published:** 1892-10-29

**Authors:** 


					Rp inr TiAiA DC Kl o C \w/ WTTTTEXTRA NURSING SUPPLEMENT^
*8 VlO V,E.   tjff IN? Per Annum, 8s. ed? post tree.
Vol. XIII.?Oct. 29th, 1892. A ijw Monthly Parts. Gel.; post free.8d,
^ I J Ditto per Annum; 8s.. post free.
No. 318. Vol. XIII.] Saturday,
October 29th, 1892.
[Twopence.

				

